# Impact of Polyphenol-Rich Nutraceuticals on Cognitive Function and Neuroprotective Biomarkers: A Randomized, Double-Blind, Placebo-Controlled Clinical Trial

**DOI:** 10.3390/nu17040601

**Published:** 2025-02-07

**Authors:** Juan Ángel Carrillo, Raúl Arcusa, Raquel Xandri-Martínez, Begoña Cerdá, Pilar Zafrilla, Javier Marhuenda

**Affiliations:** 1 Faculty of Pharmacy and Nutrition, Universidad Católica San Antonio, 30107 Murcia, Spain; jacarrillo4@alu.ucam.edu (J.Á.C.); rarcusa@ucam.edu (R.A.); bcerda@ucam.edu (B.C.);; 2 Faculty of Health Sciences, Universidad Católica de San Antonio, 30107 Murcia, Spain; rxandri@ucam.edu

**Keywords:** polyphenols, neuroprotection, BDNF (Brain-Derived Neurotrophic Factor), CREB (cAMP Response Element-Binding Protein), cognitive function, nutraceuticals, synaptic plasticity

## Abstract

Background: Recent studies have highlighted the neuroprotective effects of polyphenols, particularly their role in enhancing brain-derived neurotrophic factor (BDNF) and cAMP response element-binding protein (CREB) activity. This study aimed to evaluate the relationship between BDNF and CREB levels and cognitive performance in individuals undergoing a polyphenol-rich dietary intervention. Methods: A randomized, crossover, double-blind, placebo-controlled clinical trial was conducted with 92 participants. The intervention involved the daily intake of an encapsulated concentrate of fruit, vegetable, and berry juice powders (Juice Plus+ Premium^®^) over two 16-week periods, separated by a 4-week washout phase. Cognitive function was assessed using the Stroop Test, Trail Making Test, and Reynolds Intellectual Screening Test (RIST). The plasma levels of CREB and BDNF were measured using ELISA. Results: The polyphenol-rich product significantly improved cognitive performance, as evidenced by higher scores in the Stroop Test and RIST, compared to the placebo. Additionally, the plasma levels of CREB and BDNF were notably elevated in the product condition, indicating enhanced neuroprotective activity. Conclusions: The findings suggest that polyphenol-rich nutraceuticals can modulate neurobiological mechanisms underlying cognitive improvements, primarily through the reduction of oxidative stress and the regulation of signaling pathways associated with synaptic plasticity. These results support the potential of dietary polyphenols in promoting cognitive health and preventing neurodegenerative diseases.

## 1. Introduction

Recent research has highlighted the importance of brain proteins, such as brain-derived neurotrophic factor (BDNF) and cAMP response element-binding protein (CREB), both of which play crucial roles in synaptic plasticity, memory, and learning [1,2,3]. The activation of CREB in the hippocampus and other brain areas facilitates the expression of BDNF [4], which, subsequently, promotes neuronal survival and differentiation [5,6,7]. The evidence suggests that oxidative stress disrupts these molecular pathways, whereas antioxidant compounds like polyphenols exert neuroprotective effects by enhancing CREB and BDNF activity, thereby supporting cognitive health [8].

The scientific literature indicates that polyphenols, bioactive compounds found in fruits and vegetables, exert neuroprotective and antioxidant effects on the brain [9,10]. These compounds are capable of crossing the blood–brain barrier, where they act as modulators of signaling pathways associated with CREB and BDNF, thereby promoting improvements in synaptic plasticity and overall cognitive function [11,12,13]. In fact, we previously reported [14] that the intake of polyphenol-rich nutraceuticals reduces oxidative stress and lipid peroxidation in the central nervous system, leading to decreased neuronal damage and enhanced cognitive function [15].

Numerous studies have documented that the regular consumption of polyphenols in fruits and vegetables is associated with cognitive improvements, particularly in aspects such as memory and attention, which can be assessed through cognitive tests such as the Stroop Test, Trail Making Test, and RIST [16,17,18]. These findings support the hypothesis that a polyphenol-rich diet may contribute to the prevention of neurodegenerative diseases and the maintenance of cognitive function, even in young, healthy adults [12,19].

A unique aspect of the polyphenol-rich nutraceutical evaluated in this study lies in its composition, which integrates a blend of fruit, vegetable, and berry juice powders. This product, Juice Plus+ Premium^®^, contains over 119 distinct polyphenolic compounds, including flavanols, anthocyanins, and flavones, as demonstrated in prior compositional analyses. Compared to other polyphenol-based interventions, this nutraceutical stands out due to its comprehensive formulation, combining a wide range of bioactive compounds with complementary antioxidant and neuroprotective effects. These characteristics ensure a more diverse interaction with neurobiological pathways, including those related to oxidative stress mitigation, synaptic plasticity, and cognitive function [20].

Previous studies on similar formulations have reported significant reductions in oxidative damage and lipid peroxidation in the central nervous system, alongside improvements in cognitive performance. Such findings justify the choice of this specific product for evaluating the interplay between polyphenols, neuroprotective biomarkers, and cognitive outcomes in a healthy population [21,22].

The present study aims to evaluate the relationship between BDNF and CREB levels and cognitive performance, as measured using specific tests (Stroop Test, Trail Making Test, and RIST). By investigating this relationship, we seek to determine whether neuroprotective markers, such as BDNF and CREB, correlate with cognitive function in individuals undergoing a polyphenol-rich dietary intervention. This knowledge could enhance our understanding of the role of nutrition in cognitive health and pave the way for new preventive strategies based on dietary approaches.

## 2. Materials and Methods

### 2.1. Experimental Design

**Figure 1:**
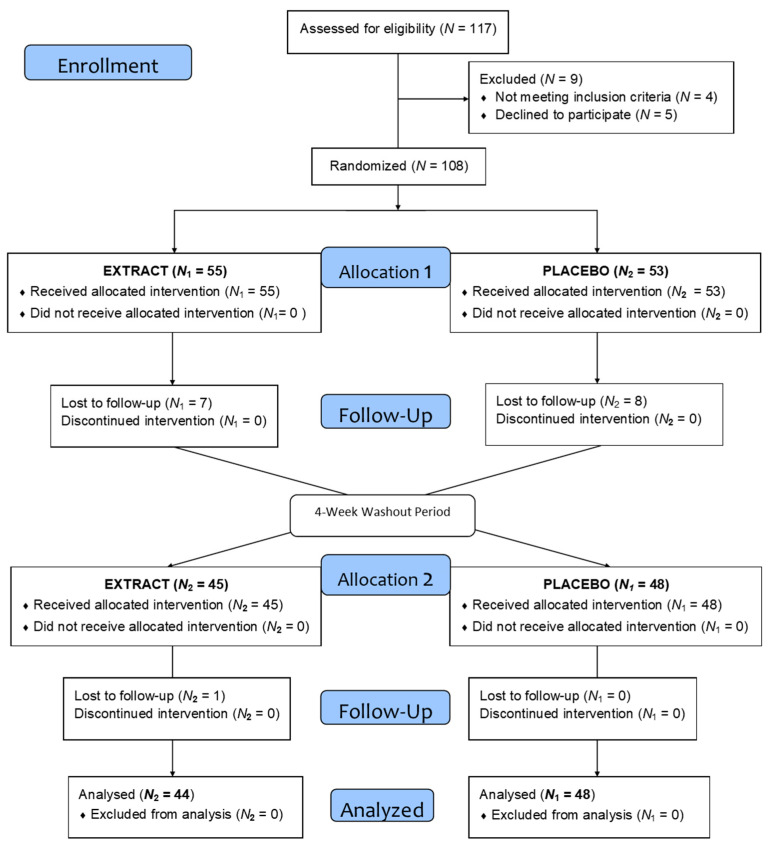
Flow diagram.

This randomized, crossover, double-blind, sex-stratified, placebo-controlled clinical trial aimed to evaluate the impact of daily intake of an encapsulated concentrate of fruit, vegetable, and berry juice powders (Juice Plus+ Premium^®^, The Juice Plus Company, Collierville, TN, USA) on cognitive functions. Participants consumed the assigned product (active or placebo) daily for two 16-week periods, separated by a 4-week washout phase. Following the washout, groups were switched, ensuring all participants received both the active product and the placebo (Figure 1).

Each participant attended a total of four visits during the study period to complete cognitive assessments (Table 1). The protocol received ethical approval from the Catholic University of Murcia (UCAM) Ethics Committee (Approval date: 24 November 2017; Code: CE111702). The study adhered to Good Clinical Practice guidelines and the principles outlined in the Declaration of Helsinki. The trial was registered at www.clinicaltrials.gov (accessed on 11 June 2020) (identifier CFE/JU/44-17). The study was carried out in the Pharmacy Department of the Faculty of Health Sciences of UCAM. Compliance with European data protection regulations (EU Regulation 2016/679) was also ensured.

### 2.2. Study Population

A total of 117 volunteers was recruited through mail distribution and posters targeting university students and staff. Of these, 108 met the initial inclusion criteria. However, 16 participants were excluded as they either failed to meet the criteria during further screening or chose to withdraw. The final sample consisted of 92 participants, comprising 47 men (51.09%) and 45 women (48.91%), with a mean age of 34 years. Table 2 provides a description of the demographic characteristics of the population.

### 2.3. Inclusion and Exclusion Criteria

Participants were required to meet the following inclusion criteria:

- Provide signed informed consent.
- Have a body mass index (BMI) between 18.5 and 35 kg/m^2^.
- Be free of chronic illnesses.
- Consume fewer than three servings of fruits or vegetables per day.
- Be aged between 18 and 65 years.

Exclusion criteria included:

- Current medical or pharmacological treatments.
- Allergies to fruits or vegetables.
- Following a special diet, vegetarianism, or veganism.
- Smoking.
- Consuming more than three alcoholic beverages (e.g., wine or beer) daily.
- Pregnancy.
- Major surgery within the past three months.
- Sleep disorders or insufficient sleep.
- Blood donation exceeding 0.5 L in the past month.

Potential confounding variables, including general nutritional intake, physical activity, and sleep patterns, were monitored through structured interviews conducted during baseline and follow-up visits to the study unit. These factors were accounted for to ensure consistency and are summarized in Table 1.

### 2.4. Tested Product

The tested product was composed of a homogenized blend of dehydrated juices and pulps from various fruits, vegetables, and berries (56%), combined in varying proportions. Key ingredients included apple, carrot, grape, pomegranate, orange, pineapple, blueberry, lingonberry, American bilberry, blackberry, cabbage, garlic, myrtle, mango, raspberry, acerola, peach, date, parsley, broccoli, spinach, kale, tomato, elderberry, blackcurrant, plum, and beet. Additional components consisted of pullulan as a coating agent, cocoa powder, green tea extract, tocopherol blend, ginger root powder, calcium ascorbate, artichoke leaf extract, grape seed extract, rice bran, spirulina powder, silicon dioxide, magnesium salts of fatty acids, citrus extract, lutein, beta-carotene, lycopene, astaxanthin, and maltodextrin. The blend was encapsulated in white pullulan capsules, with each package containing enough capsules for a 4-month consumption period. Participants were instructed to take six capsules daily (three with breakfast and three with dinner).

Polyphenolic analysis, conducted using UHPLC-QqQ-MS in a 2017 study by Bresciani et al., identified 119 distinct polyphenolic compounds in the product. These compounds spanned various phenolic families, including flavanols (e.g., kaempferol and quercetin), anthocyanins, and flavones [23]. The daily dose of six capsules provided 600 mg of a phenolic compound mixture, consistent with dosages used in other similar clinical trials [24,25].

To maintain blinding, the placebo was formulated from microcrystalline cellulose and matched the active product in both appearance and dosage, ensuring that neither participants nor researchers could distinguish between the two treatments.

### 2.5. Cognitive Assessment

#### 2.5.1. Cognitive Test

To evaluate the changes in cognitive function associated with the intervention, three cognitive tests were applied, each measuring three different domains.

The Stroop Test [26,27,28] is used to assess selective attention and inhibitory control through tasks involving reading and naming colors in congruent and incongruent contexts. The Trail Making Test (TMT) [29] evaluates processing speed, cognitive flexibility, and executive functions by alternating between connecting numbers and letters. Finally, the Reynolds Intellectual Screening Test (RIST) [30,31] is designed to provide a global measure of general cognitive abilities, assessing subcomponents such as logical reasoning and verbal recognition.

These tests allowed for the analysis of intervention effects on specific domains related to cognitive function.

#### 2.5.2. CREB and BDNF Determination

Blood samples were collected via venipuncture into EDTA-coated tubes, followed by centrifugation at 1500× *g* for 15 min at 4 °C to separate plasma. The plasma was aliquoted and stored at −80 °C until further analysis to prevent protein degradation and ensure sample integrity. This procedure is consistent with standard protocols for biomarker analysis in plasma samples [32,33]. The plasma concentrations of CREB and BDNF were measured using commercially available enzyme-linked immunosorbent assay (ELISA) kits specifically validated for human samples (Thermo fisher Scientiffic, Waltham, MA, USA) [34].

Each sample (100 µL) was analyzed in duplicate according to the manufacturer’s instructions. Plasma samples were incubated with specific antibodies conjugated to enzymes in pre-treated microplates. After incubation, detection reagents, including chromogenic substrates, were added, and absorbance was measured at 450 nm using a microplate reader [35]. Standard curves with known concentrations of CREB and BDNF were included to establish a linear relationship between absorbance and concentration, ensuring accurate quantification [36].

### 2.6. Statistical Analysis

A descriptive analysis of the quantitative study variables was conducted, presenting the mean and standard deviation (SD) for baseline conditions and their progression. The Kolmogorov–Smirnov test was employed to assess the normality of continuous data during both the experimental (EXT) and placebo (PLA) consumption periods. At baseline, comparisons between the two study arms were performed using Student’s *t*-test to ensure group homogeneity.

To evaluate changes in variables over time and between groups, a repeated measures analysis of variance (ANOVA) was conducted. This included an intrasubject factor (time: baseline and final for each study arm) and an intersubject factor (treatment: experimental product versus placebo). Post hoc analyses were carried out using the Bonferroni test to identify specific differences, with comparisons made under assumptions of both equal and unequal variances where applicable. A significance level of 0.05 was applied for all statistical tests. Data analysis was conducted using SPSS Statistics version 27 (SPSS, Inc., Chicago, IL, USA).

## 3. Results and Discussion

The evaluation of cognitive performance and neuroprotective biomarkers revealed significant differences among the three experimental conditions: baseline, placebo, and product. As summarized in Table 3, the total Stroop Test scores (PT Stroop) for Stroop W, Stroop C, and Stroop WC were consistently higher following treatment with the polyphenol-rich product (C) compared to the placebo (B) and baseline (A) conditions. The most pronounced improvement was observed in the Stroop PC task, where scores in the product group were significantly higher (*p* < 0.001) compared to the placebo (*p* = 0.04) and baseline (*p* = 0.03) groups.

Similarly, the PT RIST scores, reflecting overall cognitive abilities, demonstrated a significant increase in the product condition (*p* < 0.01) compared to the placebo and baseline conditions, indicating enhanced logical reasoning and verbal memory. Notably, this improvement correlated strongly with elevated levels of BDNF (r = 0.63; *p* < 0.001) and CREB (r = 0.55; *p* < 0.01) in the product group.

In addition, the Trail Making Test (TMT-B) performance, which assesses cognitive flexibility and executive function, showed moderate but significant positive correlations with CREB levels (r = 0.42; *p* = 0.02) in the product condition. This suggests that participants who exhibited greater increases in CREB also demonstrated faster completion times and improved task-switching abilities.

These findings align with the biochemical data, indicating that participants consuming the product experienced higher CREB levels (1.49 ± 0.28 ng/mL) compared to those consuming the placebo (1.30 ± 0.23 ng/mL) and at baseline (1.37 ± 0.21 ng/mL). Similarly, BDNF levels were significantly elevated in the product condition (7.16 ± 1.46 ng/mL) compared to the placebo (5.81 ± 1.05 ng/mL; *p* = 0.03) and baseline (6.35 ± 0.93 ng/mL; *p* = 0.01) conditions.

The correlation analysis underscores the role of BDNF and CREB as mediators in the observed cognitive enhancements, particularly in executive functions and attention tasks. These results suggest that the polyphenol-rich intervention not only improves cognitive performance, but also modulates the neurobiological pathways critical to synaptic plasticity and long-term potentiation (LTP).

In terms of neuroprotective biomarkers, CREB and BDNF levels were notably elevated in the product condition compared to the basal and placebo conditions. The group consuming the product under investigation exhibited an increase in CREB levels (1.49 ± 0.28 ng/mL) compared with the placebo group (1.30 ± 0.23 ng/mL) and baseline group (1.37 ± 0.21 ng/mL). Moreover, a notable increase in BDNF levels was observed in the product group (7.16 ± 1.46 ng/mL) compared to the placebo (5.81 ± 1.05 ng/mL) and baseline (6.35 ± 0.93 ng/mL) groups.

Comparing the placebo and product groups, the product group consistently showed greater improvements across all measured outcomes. The placebo group exhibited more modest changes, with no substantial differences compared to baseline that surpassed those observed in the product group. These results suggest that the product has a more pronounced effect on cognitive, physical, and biochemical measures than the placebo, highlighting its potential as a beneficial intervention for enhancing brain function.

The results of this study highlight a clear relationship between the consumption of the polyphenol-rich product and improvements in cognitive function and neuroprotective biomarkers, CREB and BDNF. These findings support growing evidence that polyphenols can modulate key neurobiological processes underlying cognitive improvements, primarily through the reduction of oxidative stress and the regulation of signaling pathways associated with synaptic plasticity [37,38,39].

The significant increase in CREB and BDNF levels observed in the product condition is a notable result that reinforces the impact of polyphenols on neuroprotection [40,41]. CREB is an essential transcription factor for synaptic plasticity, as it promotes the expression of BDNF and other neuroprotective genes [42]. BDNF, in turn, acts as a key modulator of neuronal plasticity, facilitating synaptic strengthening, dendritic growth, and neuronal survival [43,44]. The relationship between these biomarkers and the observed cognitive function improvements in this study suggests that the investigated product activates critical pathways for learning and memory [45,46].

In this context, it is important to highlight the observed correlation between elevated BDNF and CREB levels and the improvements in the Stroop Test and RIST scores. The Stroop Test, designed to measure selective attention and inhibitory control, showed significant improvements in the product condition, particularly in the Stroop PC task. Such tasks, requiring a high capacity for conflict resolution, are highly dependent on the functional integrity of areas such as the prefrontal cortex and hippocampus, which are both modulated by BDNF [47,48,49]. The cognitive tests employed in this study, particularly the Stroop Test and RIST, assess several key domains, including selective attention, inhibitory control, logical reasoning, and verbal memory. While memory is directly evaluated through these tests, the impact of the polyphenol-rich product on learning was not specifically assessed. Nevertheless, the significant increases in BDNF and CREB levels observed suggest potential benefits for synaptic plasticity and mechanisms underlying learning, such as long-term potentiation (LTP). Future research incorporating tests designed to measure learning explicitly would provide a more comprehensive understanding of the cognitive effects of polyphenol-rich interventions. The significant correlation between the plasma levels of BDNF and CREB and the cognitive improvements observed in this study raises the question of their causal relationship. Plasma BDNF levels have been shown to correlate with hippocampal BDNF expression, particularly in areas critical for learning and memory, such as the hippocampus and prefrontal cortex. However, peripheral biomarkers are an indirect proxy for neuronal activity, as their concentrations can be influenced by systemic factors and the permeability of the blood–brain barrier. Despite these limitations, the substantial increases in BDNF and CREB observed in this study are consistent with their established roles in synaptic plasticity and cognitive function. Future investigations combining biomarker analysis with neuroimaging assessments could further validate these findings and provide more direct insights into the brain-specific effects of dietary polyphenols. Conversely, the improvements observed in the RIST, which evaluates general cognitive skills, such as logical reasoning and verbal memory, are also consistent with the increase in BDNF and CREB levels. This supports the hypothesis that these proteins play a mediating role in the cognitive effects observed, acting as convergence points between dietary interventions and the functional responses of the central nervous system [50,51]. While this study did not identify the specific polyphenol responsible for the observed effects, evidence suggests that the combination of multiple polyphenols may exert synergistic neuroprotective effects. The diverse composition of the product likely contributes collectively to reducing oxidative stress and enhancing cognitive function. Future studies should investigate the roles of individual polyphenols to better elucidate their contributions.

The relationship between precision in executive tasks and levels of CREB and BDNF also deserves attention. Although a slight decrease in processing speed was observed in the product condition, the precision improved significantly, suggesting a possible cognitive prioritization shift towards accuracy over speed. This trend may be mediated by the effects of BDNF in stabilizing synaptic networks, optimizing the performance in tasks that require greater executive control [52,53,54]. This phenomenon is consistent with previous studies showing that polyphenol-based interventions can favor precision over speed in complex cognitive tasks, possibly due to greater efficiency in synaptic transmission [35].

Furthermore, the results related to CREB are particularly relevant, due to its role in long-term memory consolidation. CREB regulates the transcription of genes associated with the formation of new synapses, a crucial process for sustained learning [55]. The improvement observed in RIST scores and their correlation with CREB levels suggest that the product may have a specific effect on long-term potentiation (LTP), the synaptic mechanism underlying learning and memory. This finding highlights the product’s potential to influence not only immediate processes, such as attention, but also deeper mechanisms associated with memory and cognitive adaptation [56,57,58,59].

Another notable aspect of this study is the increase in BDNF levels, a molecule not only related to synaptic plasticity, but also playing a critical role in protecting against neuronal damage. The increase in BDNF levels in the product condition suggests that polyphenol consumption could contribute to the resilience of the nervous system against aging and oxidative stress, with significant clinical implications for the prevention of neurodegenerative diseases [60,61,62].

Finally, although the results are promising, certain limitations must be considered. The crossover and randomized design ensure high internal validity, but the sample, limited to healthy young adults, could restrict the generalization of findings to other populations, such as older adults or individuals with cognitive impairment. While this study was not designed to evaluate long-term efficacy, the existing evidence suggests that the sustained consumption of polyphenol-rich products could further enhance cognitive function due to the cumulative neuroprotective effects of polyphenols. Their antioxidative properties and roles in modulating synaptic plasticity and neurogenesis make it plausible that prolonged intake would continue to support neuronal resilience and cognitive performance.

Similarly, although this study focused on healthy young adults, the observed mechanisms of action, such as the elevation of BDNF and CREB levels, are relevant to populations with cognitive dysfunction. It is reasonable to hypothesize that individuals with cognitive impairments might experience comparable or even more pronounced benefits from such interventions. Future studies should investigate these effects in older populations and those with neurodegenerative conditions to validate these potential applications.

Additionally, genotypic differences among participants could influence individual responses to the polyphenol-rich product. Variations in genes associated with oxidative stress pathways, polyphenol metabolism, or neurotrophic factors such as BDNF and CREB may modulate the observed effects. Incorporating genotypic analyses in future studies could provide a more comprehensive understanding of these individual differences and their impact on cognitive outcomes. Moreover, it would be interesting to evaluate whether the observed improvements in biomarkers and cognitive tests persist over time, exploring the sustainability of the benefits associated with the product’s consumption.

Overall, this study provides solid evidence of the impact of consuming a polyphenol-rich product on improving key cognitive functions and activating neuroprotective biomarkers. These findings reinforce the potential of nutraceuticals as innovative tools for promoting brain health and open new opportunities for dietary interventions aimed at maintaining cognitive function.

## 4. Conclusions

The present study reports the effect of a polyphenol-rich nutraceutical on cognitive performance in relation to increased levels of neuroprotective biomarkers, specifically BDNF and CREB. Specifically, the improvement in the Stroop PC (*p* < 0.001) and RIST (*p* < 0.01) results was related to increased biomarker levels, underlining synaptic plasticity as the enabling mechanism for improved cognition. Inhibitory control and conflict resolution were enhanced with increased levels of BDNF, whereas logical reasoning and verbal memory were supported by increased levels of CREB.

Importantly, the neuroprotective effects suggested in the present study extend beyond immediate cognitive improvements, suggesting applications for mitigating age-related cognitive decline and preventing neurodegenerative diseases. The alignment between elevated biomarkers and improved test performance supports the hypothesis that BDNF and CREB serve as key mediators, acting as convergence points between dietary interventions and enhanced central nervous system function. The strong correlation between cognitive performance and elevated BDNF and CREB levels reinforces the transformative potential of nutraceuticals as innovative tools for maintaining brain health, facilitating neuroprotection, and opening pathways for targeted dietary interventions aimed at preserving cognitive function and preventing neurological decline.

## Figures and Tables

**Table 1:** Follow-up actions at each unit visit.

Monitoring	VS	V1	V2	V3	V4
Informed consent	X				
Inclusion/exclusion criteria	X				
History. Lifestyle, dietary, and eating habits	X				
Randomization	X				
Product/placebo delivery		X		X	
Cognition tests		X	X		X
Adherence to the study		X	X	X	X
Adverse events		X	X	X	X

V = Visits to the research unit.

**Table 2:** Demographic data.

	N_1_	N_2_	Total
N	48	44	92
Age (years)	33 ± 10	36 ± 12	34 ± 11
Women	28	19	47
Men	20	25	45
Height	1.71 ± 9	1.73 ± 9	1.72 ± 9
Weight	70.68 ± 13.88	75.68 ± 14.44	73.10 ± 14.29
BMI (kg/m^2^)	23.87 ± 3.43	24.99 ± 3.38	24.40 ± 3.43

BMI (Body mass index).

**Table 3:** Results of cognitive assessments and neuroprotective biomarkers in the three experimental conditions (mean ± SD).

Variable	Basal (A)	Placebo (B)	Product (C)
PT Stroop (W)	46.05 ± 7.63	45.72 ± 6.58	49.74 ± 7.26 *
PT Stroop (C)	49.39 ± 8.66	49.67 ± 6.40	52.46 ± 9.73 *
PT Stroop (WC)	49.40 ± 8.68	50.99 ± 7.03	53.74 ± 8.51 *
PT RIST	95.91 ± 16.24	98.03 ± 14.36	110.42 ± 11.06 *
PD Execution	19.87 ± 4.34	21.48 ± 2.41	24.91 ± 4.70 *
PD Speed	405.03 ± 57.20	394.71 ± 45.90	366.22 ± 76.81 *
PD Accuracy	97.75 ± 6.39	98.17 ± 9.39	99.23 ± 1.60 *
CREB (ng/mL)	1.37 ± 0.21	1.30 ± 0.23	1.49 ± 0.28 *
BDNF (ng/mL)	6.35 ± 0.93	5.81 ± 1.05	7.16 ± 1.46 *

* There are statistically significant differences (*p* < 0.05).

## Data Availability

The data are contained within the article.

## References

[1] Miranda M., Morici J.F., Zanoni M.B., Bekinschtein P. (2019). Brain-Derived Neurotrophic Factor: A Key Molecule for Memory in the Healthy and the Pathological Brain. Front. Cell. Neurosci..

[2] Zheng F., Zhou X., Moon C., Wang H. (2012). Regulation of Brain-Derived Neurotrophic Factor Expression in Neurons. Int. J. Physiol. Pathophysiol. Pharmacol..

[3] Poo M. (2001). Neurotrophins as Synaptic Modulators. Nat. Rev. Neurosci..

[4] Witte A.V., Kerti L., Margulies D.S., Flöel A. (2014). Effects of Resveratrol on Memory Performance, Hippocampal Functional Connectivity, and Glucose Metabolism in Healthy Older Adults. J. Neurosci..

[5] Lonze B.E., Ginty D.D. (2002). Function and Regulation of CREB Family Transcription Factors in the Nervous System. Neuron.

[6] Mattson M., Meffert M. (2006). Roles for NF-κB in Nerve Cell Survival, Plasticity, and Disease. Cell Death Differ..

[7] Tabuchi A., Sakaya H., Kisukeda T., Fushiki H., Tsuda M. (2002). Involvement of an Upstream Stimulatory Factor as Well as cAMP-Responsive Element-Binding Protein in the Activation of Brain-Derived Neurotrophic Factor Gene Promoter I. J. Biol. Chem..

[8] Scapagnini G., Davinelli S., Drago F., De Lorenzo A., Oriani G. (2012). Antioxidants as Antidepressants: Fact or Fiction?. CNS Drugs.

[9] Gómez-Pinilla F. (2008). Brain Foods: The Effects of Nutrients on Brain Function. Nat. Rev. Neurosci..

[10] Spencer J.P., Vauzour D., Rendeiro C. (2009). Flavonoids and Cognition: The Molecular Mechanisms Underlying Their Behavioural Effects. Arch. Biochem. Biophys..

[11] Vauzour D. (2012). Dietary Polyphenols as Modulators of Brain Functions: Biological Actions and Molecular Mechanisms Underpinning Their Beneficial Effects. Oxid. Med. Cell. Longev..

[12] Rendeiro C., Guerreiro J.D., Williams C.M., Spencer J.P. (2012). Flavonoids as Modulators of Memory and Learning: Molecular Interactions Resulting in Behavioural Effects. Proc. Nutr. Soc..

[13] Youdim K.A., Qaiser M.Z., Begley D.J., Rice-Evans C.A., Abbott N.J. (2004). Flavonoid Permeability across an In Situ Model of the Blood–Brain Barrier. Free Radic. Biol. Med..

[14] Arcusa R., Carillo J.Á., Cerdá B., Durand T., Gil-Izquierdo Á., Medina S., Galano J.-M., Zafrilla M.P., Marhuenda J. (2023). Ability of a Polyphenol-Rich Nutraceutical to Reduce Central Nervous System Lipid Peroxidation by Analysis of Oxylipins in Urine: A Randomized, Double-Blind, Placebo-Controlled Clinical Trial. Antioxidants.

[15] Hussain G., Zhang L., Rasul A., Anwar H., Sohail M.U., Razzaq A., Aziz N., Shabbir A., Ali M., Sun T. (2018). Role of Plant-Derived Flavonoids and Their Mechanism in Attenuation of Alzheimer’s and Parkinson’s Diseases: An Update of Recent Data. Molecules.

[16] Whyte A.R., Cheng N., Fromentin E., Williams C.M. (2018). A Randomized, Double-Blinded, Placebo-Controlled Study to Compare the Safety and Efficacy of Low Dose Enhanced Wild Blueberry Powder and Wild Blueberry Extract (ThinkBlue^TM^) in Maintenance of Episodic and Working Memory in Older Adults. Nutrients.

[17] Gardener S.L., Rainey-Smith S.R., Weinborn M., Bondonno C.P., Martins R.N. (2021). Intake of Products Containing Anthocyanins, Flavanols, and Flavanones, and Cognitive Function: A Narrative Review. Front. Aging Neurosci..

[18] Nilsson A., Salo I., Plaza M., Björck I. (2017). Effects of a Mixed Berry Beverage on Cognitive Functions and Cardiometabolic Risk Markers; A Randomized Cross-over Study in Healthy Older Adults. PLoS ONE.

[19] Rodriguez-Mateos A., Vauzour D., Krueger C.G., Shanmuganayagam D., Reed J., Calani L., Mena P., Del Rio D., Crozier A. (2014). Bioavailability, Bioactivity and Impact on Health of Dietary Flavonoids and Related Compounds: An Update. Arch. Toxicol..

[20] Carrillo J.Á., Arcusa R., Zafrilla M.P., Marhuenda J. (2021). Effects of Fruit and Vegetable-Based Nutraceutical on Cognitive Function in a Healthy Population: Placebo-Controlled, Double-Blind, and Randomized Clinical Trial. Antioxidants.

[21] Dams S., Holasek S., Tsiountsioura M., Malliga D.-E., Meier-Allard N., Poncza B., Lackner S., Jansenberger Y., Lamprecht M. (2019). An Encapsulated Fruit, Vegetable and Berry Juice Powder Concentrate Increases Plasma Values of Specific Carotenoids and Vitamins. Int. J. Vitam. Nutr. Res..

[22] Spencer J.P. (2009). Flavonoids and Brain Health: Multiple Effects Underpinned by Common Mechanisms. Genes Nutr..

[23] Bresciani L., Martini D., Mena P., Tassotti M., Calani L., Brigati G., Brighenti F., Holasek S., Malliga D.-E., Lamprecht M. (2017). Absorption Profile of (Poly) Phenolic Compounds after Consumption of Three Food Supplements Containing 36 Different Fruits, Vegetables, and Berries. Nutrients.

[24] Novembrino C., Cighetti G., De Giuseppe R., Vigna L., de Liso F., Pellegatta M., Gregori D., Maiavacca R., Bamonti F. (2011). Effects of Encapsulated Fruit and Vegetable Juice Powder Concentrates on Oxidative Status in Heavy Smokers. J. Am. Coll. Nutr..

[25] Lamprecht M., Oettl K., Schwaberger G., Hofmann P., Greilberger J.F. (2007). Several Indicators of Oxidative Stress, Immunity, and Illness Improved in Trained Men Consuming an Encapsulated Juice Powder Concentrate for 28 Weeks, 3. J. Nutr..

[26] Golden C., Freshwater S.M., Golden Z. (1978). Stroop Color and Word Test. https://psycnet.apa.org/doiLanding?doi=10.1037%2Ft06065-000.

[27] Whyte A.R., Williams C.M. (2015). Effects of a Single Dose of a Flavonoid-Rich Blueberry Drink on Memory in 8 to 10 y Old Children. Nutrition.

[28] Carillon J., Notin C., Schmitt K., Simoneau G., Lacan D. (2014). Dietary Supplementation with a Superoxide Dismutase-Melon Concentrate Reduces Stress, Physical and Mental Fatigue in Healthy People: A Randomised, Double-Blind, Placebo-Controlled Trial. Nutrients.

[29] Portellano J., Martínez Arias R.T. (2014). Test de Los Senderos.

[30] Raines T.C., Reynolds C.R., Kamphaus R.W. (2018). The Reynolds Intellectual Assessment Scales, and the Reynolds Intellectual Screening Test. Contemporary Intellectual Assessment: Theories, Tests, and Issues.

[31] Greve A., Jepsen J.R.M., Mortensen E.L., Uher R., Mackenzie L., Foldager L., Gantriis D., Burton B.K., Ellersgaard D., Christiani C.J. (2018). F84. Associations between Intelligence, Verbal Working Memory and Processing Speed in Parents with Schizophrenia or Bipolar Disorder and Their 7-Year Old Offspring. Schizophr. Bull..

[32] Lee B.-H., Kim H., Park S.-H., Kim Y.-K. (2007). Decreased Plasma BDNF Level in Depressive Patients. J. Affect. Disord..

[33] Koch J.M., Hinze-Selch D., Stingele K., Huchzermeier C., Göder R., Seeck-Hirschner M., Aldenhoff J.B. (2009). Changes in CREB Phosphorylation and BDNF Plasma Levels during Psychotherapy of Depression. Psychother. Psychosom..

[34] Habibi P., Shahidi S., Khajvand-Abedini M., Shahabi Z., Ahmadiasl N., Alipour M.R., Ramezani M., Komaki A. (2024). Effect of Young Plasma Therapy on Cognition, Oxidative Stress, miRNA-134, BDNF, CREB, and SIRT-1 Expressions and Neuronal Survey in the Hippocampus of Aged Ovariectomized Rats with Alzheimer’s. Brain Sci..

[35] Huang R., Lin Y., Shi Q., Flowers L., Ramachandran S., Horowitz I.R., Parthasarathy S., Huang R.-P. (2004). Enhanced Protein Profiling Arrays with ELISA-Based Amplification for High-Throughput Molecular Changes of Tumor Patients’ Plasma. Clin. Cancer Res..

[36] Lin D., Alborn W.E., Slebos R.J., Liebler D.C. (2013). Comparison of Protein Immunoprecipitation-Multiple Reaction Monitoring with ELISA for Assay of Biomarker Candidates in Plasma. J. Proteome Res..

[37] Naomi R., Yazid M.D., Teoh S.H., Balan S.S., Shariff H., Kumar J., Bahari H., Embong H. (2023). Dietary Polyphenols as a Protection against Cognitive Decline: Evidence from Animal Experiments; Mechanisms and Limitations. Antioxidants.

[38] Mekhora C., Lamport D.J., Spencer J.P. (2024). Effect of Polyphenols on Inflammation Related to Cognitive Function: A Systematic Review and Meta-Analysis of Human Randomized Controlled Trials. Nutr. Healthy Aging.

[39] Arias-Sánchez R.A., Torner L., Fenton Navarro B. (2023). Polyphenols and Neurodegenerative Diseases: Potential Effects and Mechanisms of Neuroprotection. Molecules.

[40] Tavan M., Hanachi P., de la Luz Cádiz-Gurrea M., Segura Carretero A., Mirjalili M.H. (2024). Natural Phenolic Compounds with Neuroprotective Effects. Neurochem. Res..

[41] Schaffer S., Asseburg H., Kuntz S., Muller W.E., Eckert G.P. (2012). Effects of Polyphenols on Brain Ageing and Alzheimer’s Disease: Focus on Mitochondria. Mol. Neurobiol..

[42] Zhang S., Xue R., Hu R. (2020). The Neuroprotective Effect and Action Mechanism of Polyphenols in Diabetes Mellitus-Related Cognitive Dysfunction. Eur. J. Nutr..

[43] Kowiański P., Lietzau G., Czuba E., Waśkow M., Steliga A., Moryś J. (2018). BDNF: A Key Factor with Multipotent Impact on Brain Signaling and Synaptic Plasticity. Cell. Mol. Neurobiol..

[44] De Vincenti A.P., Ríos A.S., Paratcha G., Ledda F. (2019). Mechanisms That Modulate and Diversify BDNF Functions: Implications for Hippocampal Synaptic Plasticity. Front. Cell. Neurosci..

[45] Colucci-D’Amato L., Speranza L., Volpicelli F. (2020). Neurotrophic Factor BDNF, Physiological Functions and Therapeutic Potential in Depression, Neurodegeneration and Brain Cancer. Int. J. Mol. Sci..

[46] Ortega-Martínez S. (2015). A New Perspective on the Role of the CREB Family of Transcription Factors in Memory Consolidation via Adult Hippocampal Neurogenesis. Front. Mol. Neurosci..

[47] Jaberi S., Fahnestock M. (2023). Mechanisms of the Beneficial Effects of Exercise on Brain-Derived Neurotrophic Factor Expression in Alzheimer’s Disease. Biomolecules.

[48] Tarassova O., Ekblom M.M., Moberg M., Lövdén M., Nilsson J. (2020). Peripheral BDNF Response to Physical and Cognitive Exercise and Its Association with Cardiorespiratory Fitness in Healthy Older Adults. Front. Physiol..

[49] Lu B., Nagappan G., Lu Y. (2014). BDNF and Synaptic Plasticity, Cognitive Function, and Dysfunction. Neurotrophic Factors.

[50] Brigadski T., Leßmann V. (2014). BDNF: A Regulator of Learning and Memory Processes with Clinical Potential. e-Neuroforum.

[51] Heberden C. (2016). Modulating Adult Neurogenesis through Dietary Interventions. Nutr. Res. Rev..

[52] Moya-Alvarado G., Tiburcio-Felix R., Ibáñez M.R., Aguirre-Soto A.A., Guerra M.V., Wu C., Mobley W.C., Perlson E., Bronfman F.C. (2023). BDNF/TrkB Signaling Endosomes in Axons Coordinate CREB/mTOR Activation and Protein Synthesis in the Cell Body to Induce Dendritic Growth in Cortical Neurons. eLife.

[53] Leckie R.L., Oberlin L.E., Voss M.W., Prakash R.S., Szabo-Reed A., Chaddock-Heyman L., Phillips S.M., Gothe N.P., Mailey E., Vieira-Potter V.J. (2014). BDNF Mediates Improvements in Executive Function Following a 1-Year Exercise Intervention. Front. Hum. Neurosci..

[54] Esvald E.-E., Tuvikene J., Sirp A., Patil S., Bramham C.R., Timmusk T. (2020). CREB Family Transcription Factors Are Major Mediators of BDNF Transcriptional Autoregulation in Cortical Neurons. J. Neurosci..

[55] Kandel E.R. (2012). The Molecular Biology of Memory: cAMP, PKA, CRE, CREB-1, CREB-2, and CPEB. Mol. Brain.

[56] Suzuki A., Fukushima H., Mukawa T., Toyoda H., Wu L.-J., Zhao M.-G., Xu H., Shang Y., Endoh K., Iwamoto T. (2011). Upregulation of CREB-Mediated Transcription Enhances Both Short-and Long-Term Memory. J. Neurosci..

[57] Gandolfi D., Cerri S., Mapelli J., Polimeni M., Tritto S., Fuzzati-Armentero M.-T., Bigiani A., Blandini F., Mapelli L., D’Angelo E. (2017). Activation of the CREB/c-Fos Pathway during Long-Term Synaptic Plasticity in the Cerebellum Granular Layer. Front. Cell. Neurosci..

[58] Gruart A., Benito E., Delgado-García J.M., Barco A. (2012). Enhanced cAMP Response Element-Binding Protein Activity Increases Neuronal Excitability, Hippocampal Long-Term Potentiation, and Classical Eyeblink Conditioning in Alert Behaving Mice. J. Neurosci..

[59] Scott R., Bourtchuladze R., Gossweiler S., Dubnau J., Tully T. (2002). CREB and the Discovery of Cognitive Enhancers. J. Mol. Neurosci..

[60] Annunziata G., Sureda A., Orhan I.E., Battino M., Arnone A., Jiménez-García M., Capo X., Cabot J., Sanadgol N., Giampieri F. (2021). The Neuroprotective Effects of Polyphenols, Their Role in Innate Immunity and the Interplay with the Microbiota. Neurosci. Biobehav. Rev..

[61] Grabska-Kobyłecka I., Szpakowski P., Król A., Książek-Winiarek D., Kobyłecki A., Głąbiński A., Nowak D. (2023). Polyphenols and Their Impact on the Prevention of Neurodegenerative Diseases and Development. Nutrients.

[62] Li Z., Zhao T., Shi M., Wei Y., Huang X., Shen J., Zhang X., Xie Z., Huang P., Yuan K. (2023). Polyphenols: Natural Food Grade Biomolecules for Treating Neurodegenerative Diseases from a Multi-Target Perspective. Front. Nutr..

